# Dengue NS1 antigen kit shows high sensitivity for detection of recombinant dengue virus-2 NS1 antigen spiked with *Aedes aegypti* mosquitoes

**DOI:** 10.1038/s41598-021-02965-x

**Published:** 2021-12-08

**Authors:** Philip Raj Abraham, Bharathy R, Pradeep Kumar N, Ashwani Kumar

**Affiliations:** 1 Unit of Omics, ICMR-Vector Control Research Centre, Puducherry, 605006 India; 2 ICMR-Vector Control Research Centre Field Station, Kottayam, 686002 Kerala India; 3 ICMR-Vector Control Research Centre, Indira Nagar, Puducherry, 605006 India; 4 Unit of Molecular Epidemiology, ICMR-Vector Control Research Centre, Puducherry, 605006 India

**Keywords:** Biological techniques, Immunology, Microbiology, Biomarkers

## Abstract

Dengue, caused by the dengue virus (DENV) is a significant vector-borne disease. In absence of a specific treatment and vaccine, dengue is becoming a rising threat to public health. Currently, control of dengue mainly focuses on the surveillance of the mosquito vectors. Improved surveillance methods for DENV in mosquito populations would be highly beneficial to the public health. However, current methods of DENV detection in mosquitoes requires specialized equipment and expensive reagents and highly trained personnel. As an alternative, commercially available dengue NS1 antigen ELISA kits could be used for detection of DENV infection in *Aedes aegypti* mosquitoes. In this study, we explored the utility of commercially available Dengue NS1 antigen kit (J. Mitra & Co. Pvt. Ltd) for the detection of recombinant dengue virus-2 (rDENV-2) NS1 protein and serum of dengue infected patient spiked with *Ae. aegypti* mosquito pools. The kit was found to be highly sensitive and specific towards detection of all serotypes of DENV. Further, it could detect as low as 750 femto gram rDENV-2 NS1 protein. It was also observed that rDENV-2 NS1 antigen spiked with blood-fed and unfed mosquito pools could be detected. In addition, the kit also detected dengue infected patient serum spiked with *Ae. aegypti* mosquito pools. Overall, the Dengue NS1 antigen kit displayed high sensitivity towards detection of recombinant as well as serum NS1 protein spiked with *Ae. aegypti* mosquito pools and could be considered for the dengue virus surveillance after a field evaluation in *Ae. aegypti* mosquitoes.

## Introduction

Dengue, a mosquito-borne viral disease is one of the serious public health problems globally. It has been estimated that dengue infection ranges from 100 to 400 million worldwide annually and 70% of the disease burden occurs mainly in Asia^1,2^. Dengue virus (DENV) is a member of the Flaviviridae family belonging to the genus Flavivirus. It is transmitted to humans through the common mosquito vectors, *Aedes aegypti* and *Aedes albopictus*^3^. Four serologically and genetically distinct serotypes namely, DENV-1, DENV-2, DENV-3 and DENV-4 can cause dengue^4^. DENV is an enveloped virus with a single positive-strand RNA genome that encodes for three structural proteins (capsid [C] protein -; pre-membrane [prM] protein—and envelope [E] protein) and seven non-structural proteins (NS1, NS2A, NS2B, NS3, NS4A, NS4B, and NS5)^4^. Among the non-structural (NS) proteins, NS1 is of great scientific interest as it plays pathogenic roles in haemorrhage and vascular leakage in dengue patients^5^. The protein is found at different cellular locations as cell surface-bound, membrane-associated, or secreted form. The secreted form of NS1 is present in the patients’ sera from the onset of illness at a concentration of 50 µg/ml sera^6,7^. These properties of NS1 lead to the development of an array of commercial rapid dengue diagnostic tests.

In absence of specific therapy and vaccines, prevention of dengue virus is mainly focused on vector management strategies. Improvements in vector surveillance methods for DENV in wild mosquito populations would be highly beneficial to public health. Nevertheless, prevention of epidemic dengue in endemic countries or prevention of its introduction into uninfested regions is very difficult with the current vector surveillance systems and control strategies^8^. To guide vector control measures, there was a call from the World Health Organization for development of integrated national vector surveillance and health information systems^9^. A positive association between dengue virus infection in human subjects and mosquitoes has been observed. Dengue was reported in human cases about a week after the manifestation of DENV-positive *Ae. aegypti* mosquitoes^10,11^. Thus, monitoring the DENV infection in *Ae. aegypti* mosquito vectors will help in risk analysis and initiating vector prevention methods using rapid DENV diagnostic tools. Indeed, surveillance of DENV prevalence in wild mosquito populations is imperative for detection of changes in vector abundance, obtaining spatial and temporal information of vector populations, evaluation of vector control programmes, as well as facilitation of appropriate evidence-based interventions^12^. In addition, surveillance of DENV prevalence in natural mosquito populations can help in preventing the onset of dengue outbreaks and implementation of effective vector control measures for instance habitat destruction, source reduction, adulticiding, and also community participation to limit possible outbreak especially in dengue endemic areas^13–15^.

Current methods of DENV detection in the field-caught mosquitoes follows tedious procedures such as isolation and propagation of virus in cell culture system and finally detection by ELISA or viral RNA extraction from the mosquitoes and confirmation using RT-PCR. These methods suffer from foremost limitations such as highly trained personnel and the need of specialized equipments. In addition, these methods are time consuming, expensive and are not feasible in developing regions of the world. In most of the countries such facilities are located in centralized facilities that require coordinated collection of mosquitoes and transport for further processing^16^. Alternatively, commercial NS1 antigen-based ELISA kits can be explored for detection of DENV-NS1 antigen in the filed-caught mosquitoes. Previously, dengue NS1 ELISA kits such as dengue early ELISA kit (Panbio), Platelia Dengue NS1 Ag (BioRad Labs) and SD Bioline NS1 Ag kit (Standards Diagnostics, Korea) have been tested for detection of DENV infection in *Ae. aegypti*^14,17,18^. In the current study, we have investigated Dengue NS1 Ag Microlisa kit (J. Mitra & Co. Pvt. Ltd, India) for detection of dengue NS1 antigen in *Ae. aegypti* mosquito population.

## Materials and methods

All methods were performed in accordance with the relevant guidelines and regulations. After receiving the Dengue NS1 Ag Microlisa kit, the purity and integrity of control proteins were checked by sodium dodecyl sulfate polyacrylamide gel electrophoresis (SDS-PAGE; 12%) following standard protocol^19^. In brief, protein samples (20 µl) were mixed with separating gel buffer and dissolved properly. After adding sample loading buffer (0.1% bromophenol blue containing SDS, glycerol and β-mercaptoethanol), the samples were incubated at 90 °C for 10 min. These sample preparations were loaded in 12% polyacrylamide gel. After electrophoresis, the gel was stained with Coomassie Brilliant blue R-250 (0.15%) for 2 h and de-stained overnight. Similarly, the NS1 antigens of other flaviviruses were also assessed on 12% SDS-PAGE.

### Determination of minimum detection limit of Dengue NS1 Ag Microlisa kit for detection of recombinant NS1 antigen

The detection limit of the Dengue NS1 Ag Microlisa kit was determined using recombinant dengue virus-2 (rDENV-2) NS1 antigen (Recombinant Viral Dengue Virus 2 NS1 Protein, CF Catalog No. 9439-DG R&D Systems, Inc. Canada). The protein concentration of rDENV-2 NS1 antigen was estimated using Bicinchoninic acid kit (Micro BCA protein assay kit from Thermo Scientific, Rockford, IL, USA). The recombinant protein was reconstituted in phosphate-buffered saline (PBS) as per the manufacturers’ instructions. Two stocks (750 picogram and 250 picogram) of rDENV-2 were prepared in 0.5 M PBS. From these stocks, subsequent dilutions were prepared (750 pg to 75 pg, 7.5 pg and 750 fg and from 250 to 25 pg, 2.5 pg and 250 fg respectively). The preparations were tested using the Dengue NS1 Ag Microlisa kit as per the manufacturer's instructions. In brief, 50 µl of diluent was added to each well followed by 50 µl of kit controls (negative control, positive control, and calibrator control) and recombinant dengue virus-2 NS1 antigen. Later, 100 µl of working conjugate was added and the Microlisa plate was incubated for 90 min at 37 °C. After washing the wells, working substrate (150 µl) was added to each well and incubated further. After adding stop solution, the absorbance was read at 450 nm in the ELISA Reader (Thermo scientific). The assay was considered valid only if the optical density (OD) of negative control was < 0.3, positive control > 1.0 and mean calibrator OD ≥ 0.35. From the sample OD, the dengue NS1 antigen units were calculated and result was interpreted as negative when dengue NS1 antigen units are < 9, equivocal if the dengue NS1 antigen units are between 9–11 and positive when the antigen units are > 11.

### Detection of sensitivity and specificity of the Dengue NS1 Ag Microlisa kit

To determine the sensitivity of the Dengue NS1 Ag Microlisa kit, recombinant NS1 antigen of all four DENV serotypes (DENV-1 to 4) were tested (The Native antigen company, Oxfordshire, UK. SKU: REC31778 to 1781). In this experiment, three concentrations (75 pg, 7.5 pg and 750 fg) of recombinant NS1 antigen were tested as per the standardized protocol of the kit. To determine the specificity, recombinant NS1 of flaviviruses such as, Japanese encephalitis virus, Tick-borne encephalitis virus, West Nile virus and Yellow fever virus (The Native antigen company, Oxfordshire, UK. SKU: FLAVX4-NS1-100) were tested at 750 pg, 250 pg and 75 pg. The antigen unit for each concentration is calculated, and the result was interpreted in terms of dengue NS1 antigen units.

### Detection of rDENV-2 NS1 antigen in *Ae. aegypti* mosquito pools

**Figure 1:**
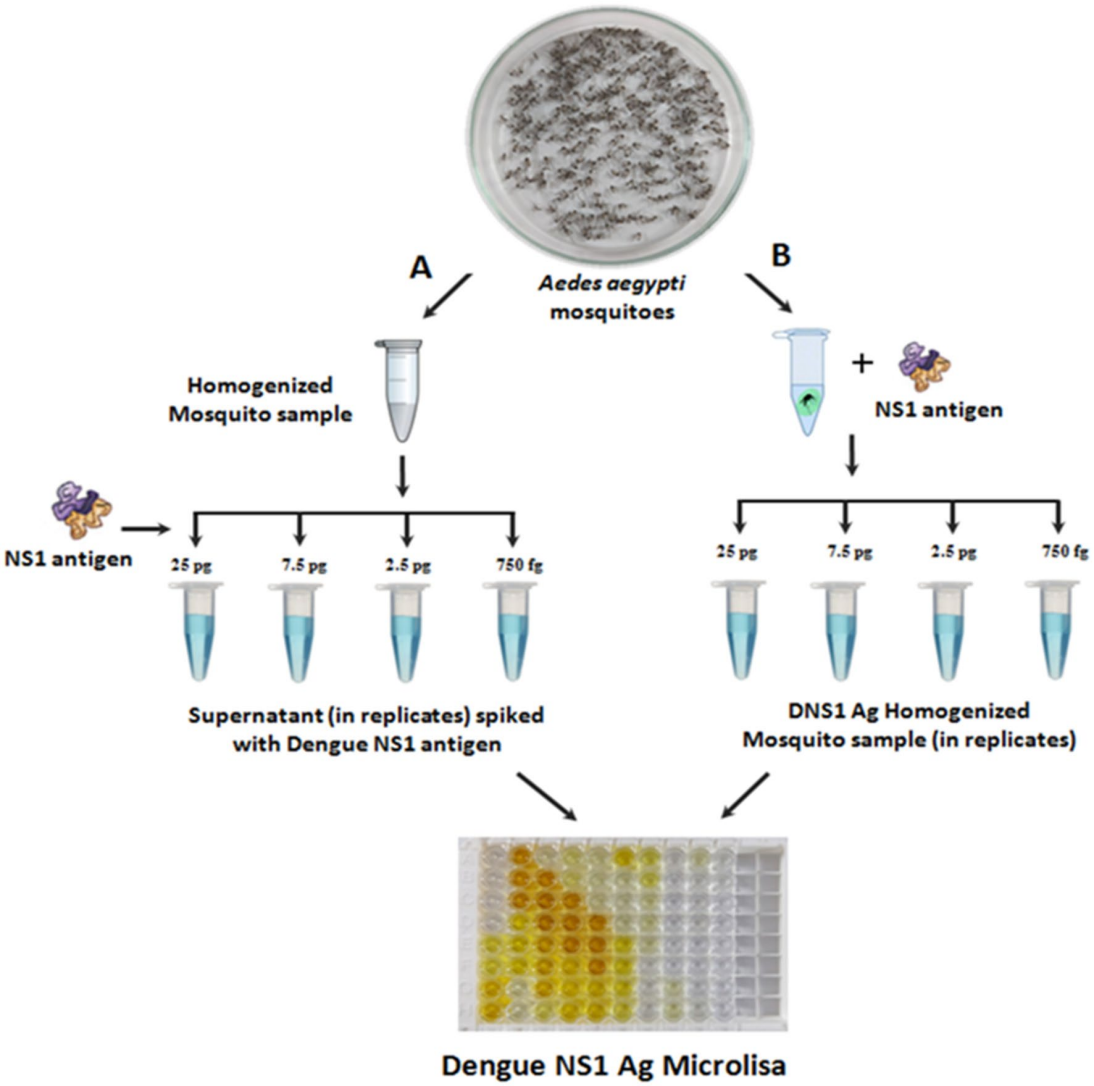
Design of spiking experiment. (**A**) The rDENV-2 NS1 antigen was added to the mosquito homogenate or (**B**) the mosquitoes were homogenized with rDENV-2 antigen and ELISA was performed.

The spiking experiment was carried out to check whether the kit can detect the rDENV-2 NS1 antigen in the mosquito pools. For this, *Ae. aegypti* mosquitoes were collected from the Rearing and Colonization laboratory of ICMR-VCRC, Puducherry. In this experiment, 25 mosquitoes per pool were homogenized following two procedures (Fig. 1). In the 1^st^ procedure (A), the mosquitoes were homogenized in a Tissue Lyser II (Qiagen, Germany) at 24 frequencies for 5 min and centrifuged at 5000 rpm (5 min.). Recombinant dengue NS1 antigen (25 pg to 750 fg) was spiked in the mosquito homogenate and ELISA was carried out. In the 2^nd^ procedure (B), the mosquitoes were spiked with the recombinant NS1 antigen at different concentrations (25 pg to 750 fg), homogenized and afterwards ELISA was performed following manufacturer's protocol. Both the procedures were carried out in replicates.

### Detection of rDENV-NS1 antigen in blood-fed *Ae. aegypti* mosquitoes

This experiment was carried out to check whether the kit can detect the recombinant NS1 antigen in the blood-fed *Ae. aegypti* mosquito pools. Based on the results of the previous experiment, two concentrations of recombinant NS1 antigen (7.5 pg and 25 pg) were spiked with pools of 25 blood-fed and unfed homogenized mosquitoes. The homogenate was tested using the Dengue NS1 Ag Microlisa kit.

### Detection of NS1 antigen of dengue infected human serum in* Ae. aegypti* mosquito pools

For collection of serum sample from the DENV infected patient, approval from the Institutional Human Ethics Committee (ECR 681/Inst/Py/2014/RR-17) of ICMR-Vector Control Research Centre, Puducherry was obtained. The patients enrolled for the study has signed an informed written consent. To simulate the condition of detection of secreted NS1 protein in the mosquitoes, serum of the DENV infected patient was spiked with *Ae. aegypti* mosquito pools. Initially, the dengue positive patient’s sera samples (50 µl) tested by the kit to confirm the presence of NS1 antigen displayed OD > 6. Therefore, the sera samples were further diluted (1:50 and 1:100) in PBS and re-tested. Both the dilutions showed OD > 6 again. Based on this, homogenized *Ae. aegypti* mosquitoes (10 and 25 mosquitoes per pool) were spiked with both dilutions of serum (1:50 and 1:100 in PBS) and tested using the kit.

## Results

### Dengue NS1 Ag Microlisa kit detects rDENV-2 NS1 antigen at low concentration

**Figure 2:**
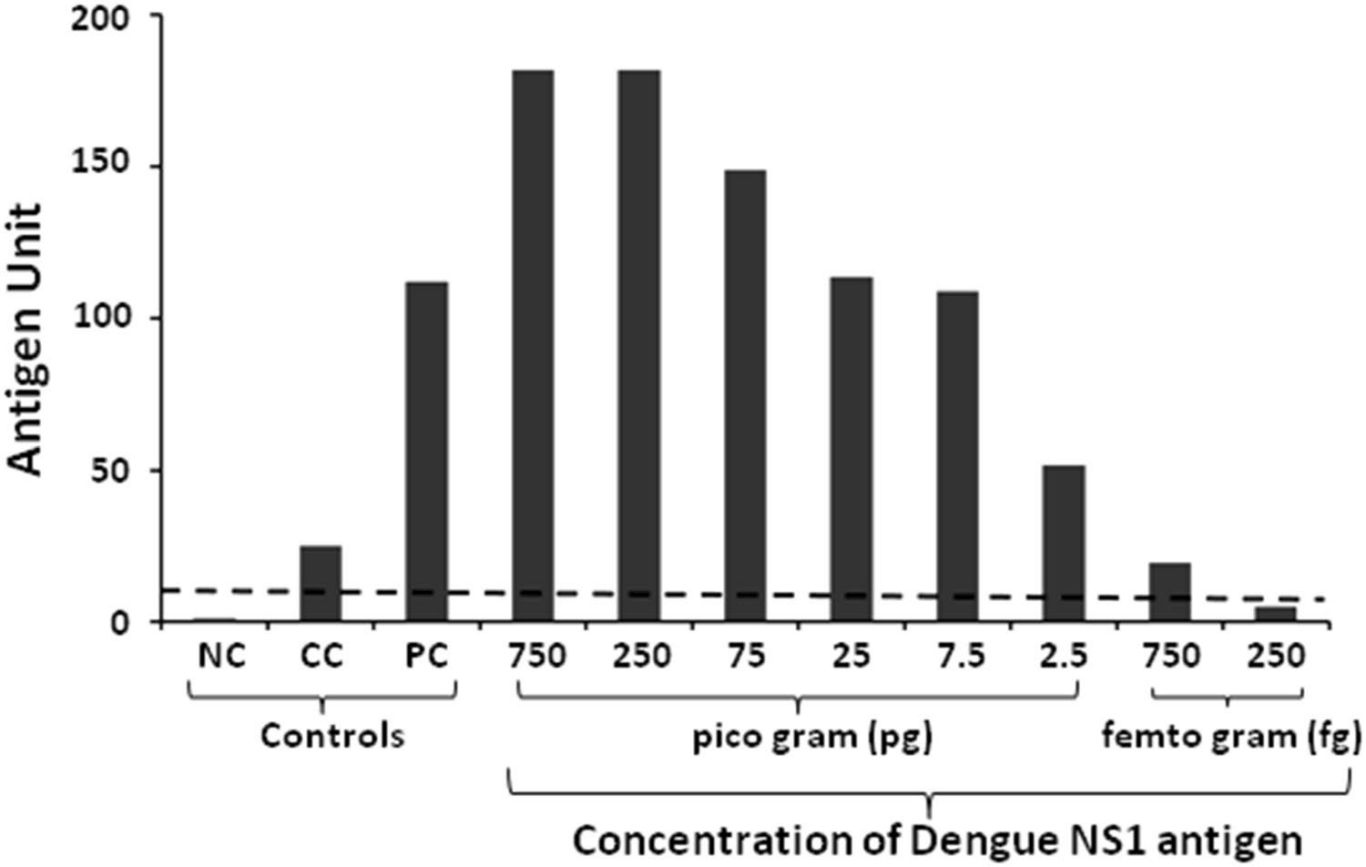
Determination of rDENV-2 NS1 detection limit of dengue NS1 Ag Microlisa kit. Recombinant Dengue NS1 antigen at different concentration was added to the wells of Microlisa plate and the assay was performed as per the instructions of the manufacturer. Absorbance was read at 450 nm in ELISA reader. *NC* Negative control, *CC* Calibrator control, *PC* Positive control. Dotted horizontal line indicates the cut-off value.

The purity and integrity of Dengue NS1 Ag Microlisa kit checked on 12% SDS-PAGE indicated that the positive control, calibrator control and enzyme conjugate were pure and intact (Fig S1). The kit displayed higher OD values (> 6) for rDENV-2 NS1 tested at 750 pg and 250 pg which was beyond the detection limit of microplate ELISA reader. After converting the OD values into antigen units, it was observed that the kit is highly sensitive to detect rDENV-2 NS1 antigen as it could detect as low as 750 fg of rDENV-2 NS1 antigen (Fig. 2).

### Dengue NS1 Ag Microlisa kit is sensitive to detect all serotypes of dengue

**Figure 3:**
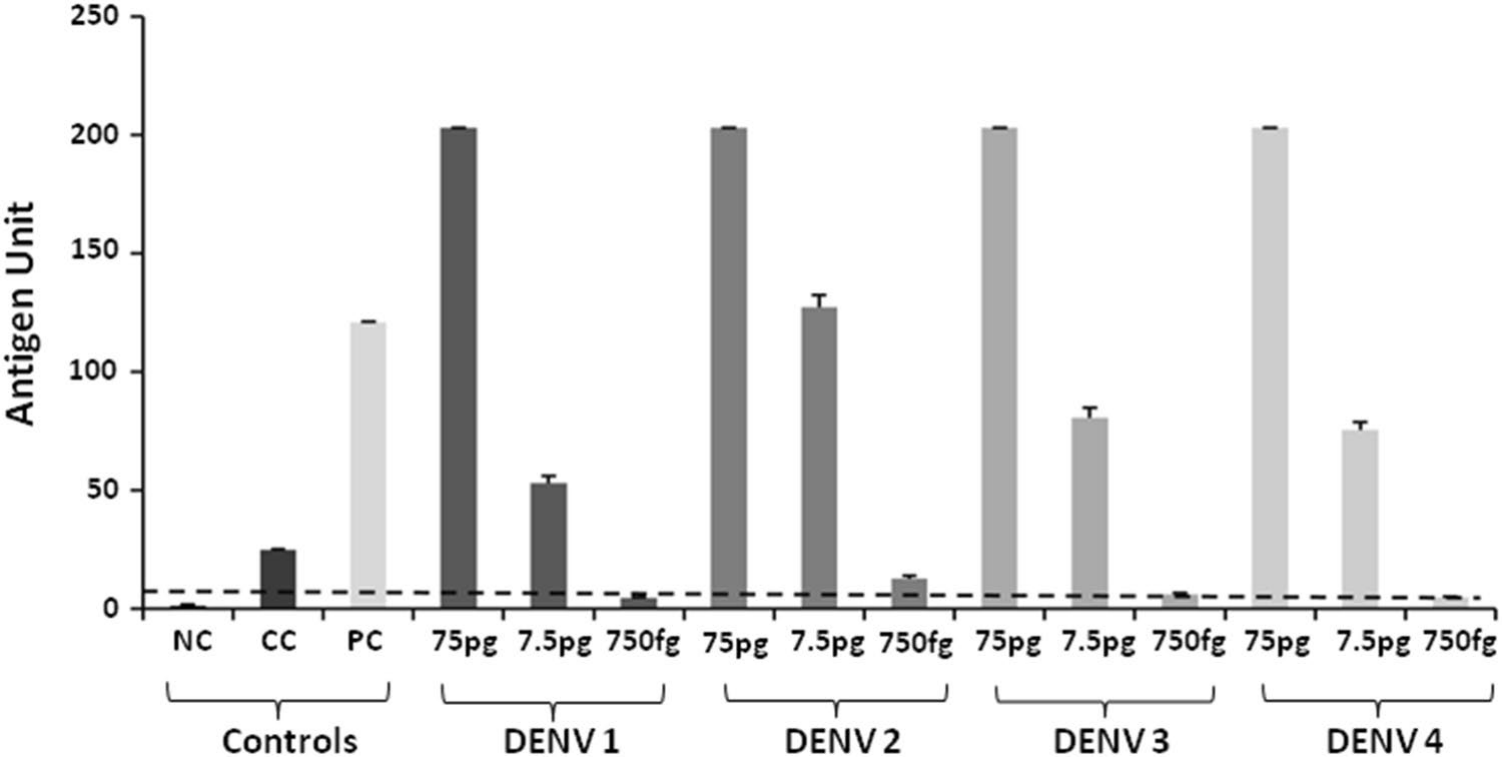
Detection of NS1 antigen of dengue serotypes. Recombinant Dengue NS1 antigens from all serotypes of dengue (DENV1 to DENV4) were tested at different concentration following manufacturers’ instructions. Cut-off value is indicated by a horizontal line. *NC* Negative control, *CC* Calibrator control, *PC* Positive control, *DENV* Dengue virus.

The NS1 antigens of flaviviruses checked on SDS-PAGE indicated that the proteins were pure and intact (Fig S2). The kit detected all the DENV serotypes suggesting that it is highly sensitive for detecting any serotype of DENV (Fig. 3). However, it was observed that the antigen units of the DENV2 were comparatively higher than the other serotypes (at 7.5 pg and 750 fg respectively). It was also observed that the detection of rDENV-2 NS1 increased with increased antigen concentration.

### Dengue NS1 Microlisa kit is highly specific for detection of only dengue NS1 antigen

**Figure 4:**
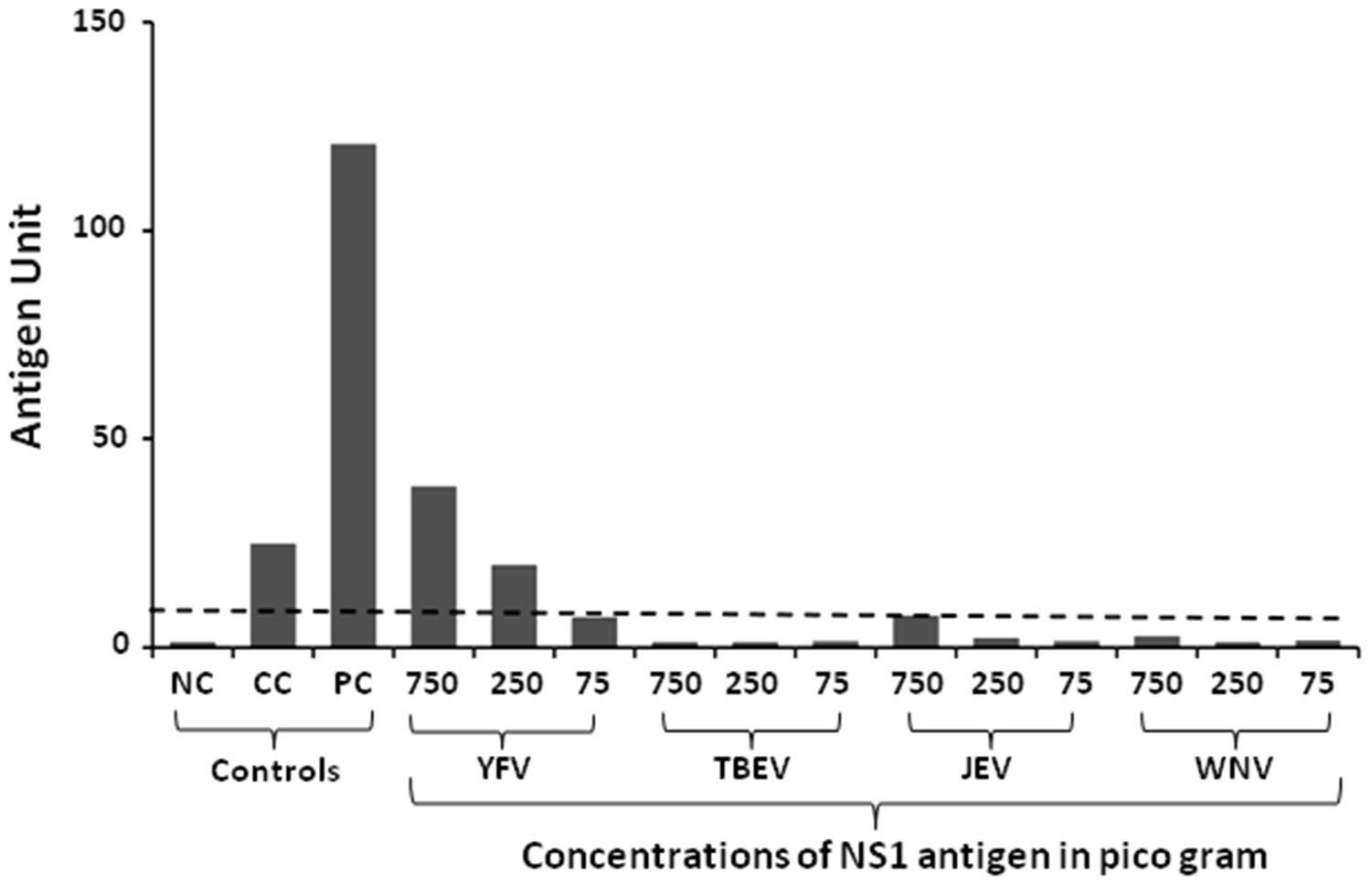
Detection of NS1 antigen of flaviviruses other than dengue serotypes. Recombinant Dengue NS1 antigen from the flaviviruses other than dengue was tested at different concentrations. *YFV* Yellow fever virus, *TBEV* Tick-borne encephalitis virus, *JEV* Japanese encephalitis virus, *WNV* West Nile virus, *NC* Negative control, *CC* Calibrator control, *PC* Positive control. Dotted horizontal line indicates the cut-off value.

Based on our previous experiment that the kit is highly sensitive to detect all the serotypes of dengue, we further determined the specificity of the kit using the recombinant NS1 antigen of flaviviruses such as yellow fever virus (YFV), tick-borne encephalitis virus (TBEV), Japanese encephalitis (JE) and West Nile virus (WNV) (Fig. 4). Only YFV showed positive results (antigen units > 11) for 750 pg and 250 pg antigen concentrations but not for 75 pg (antigen unit is 38.9, 19.9 and 7.3 respectively). In contrary, NS1 antigens of TBEV, JEV, and WNV could not show antigen units even at 750 pg antigen concentrations (antigen units < 9). This highlights that the kit is highly specific for detection of dengue NS1 only.

### Dengue NS1 Microlisa kit is sensitive to detect rDENV-2 NS1 antigen in *Ae. aegypti* mosquito pools

**Figure 5:**
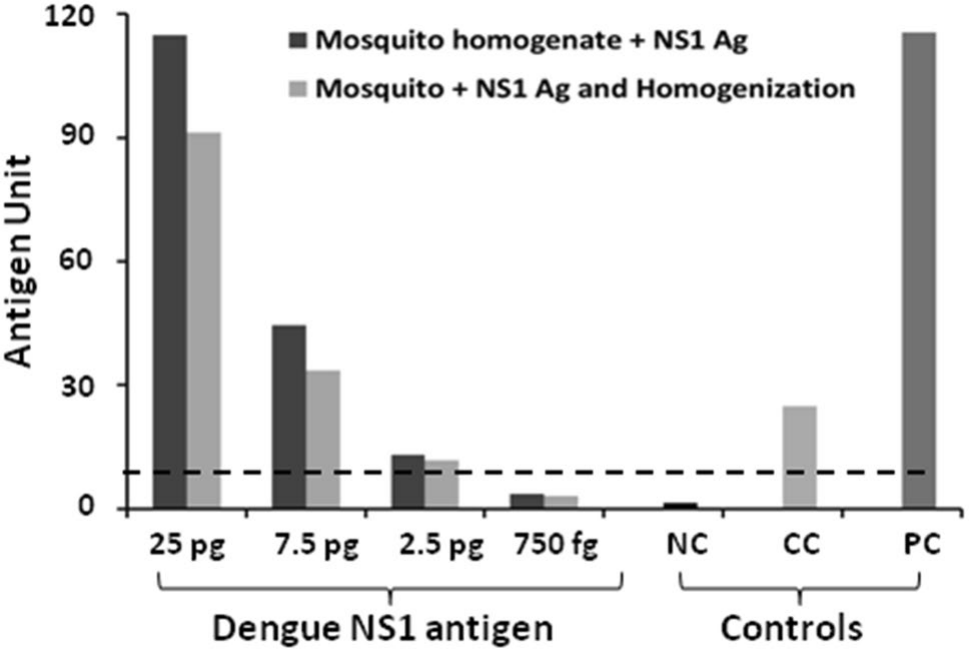
Detection of rDENV-2 NS1 antigen in *Aedes aegypti* mosquito pools. The mosquitoes were either homogenized with different concentrations of rDENV-2 NS1 or the antigen was added to homogenized mosquito samples and ELISA was performed. Antigen units were calculated as described earlier. *NC* Negative control, *CC* Calibrator control, *PC* Positive control.

This study was carried out to check whether rDENV-2 NS1 antigen spiked with *Ae. aegypti* mosquitoes can be detected using the Microlisa kit. It was observed that the kit is sensitive to detect rDENV-2 NS1 antigen in the mosquito pools up to 2.5 pg by both the procedures (Fig. 5). However, it was noteworthy that the antigen units of rDENV-2 NS1 in the mosquito pools decreased with decreased antigen concentration and none of the procedure could detect rDENV-2 NS1 antigen at 750 fg concentration.

### Dengue NS1 Microlisa kit is sensitive to detect rDENV-2 NS1 antigen in blood-fed *Ae. aegypti* mosquito pools

**Figure 6:**
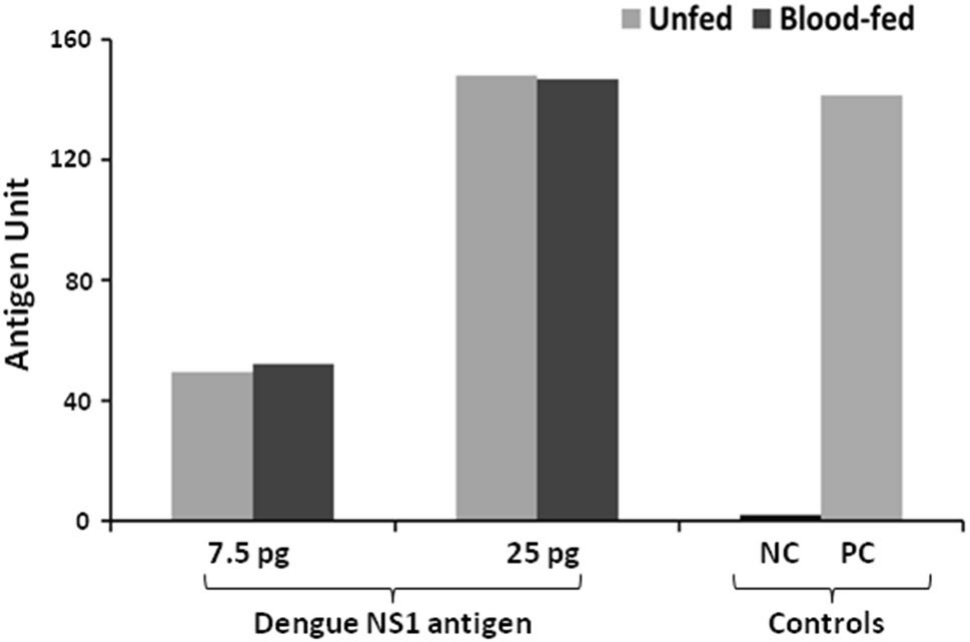
Determination of sensitivity of Microlisa kit for detection of rDENV-2 in blood fed *Ae. aegypti* mosquito pools. The mosquito homogenates were spiked with recombinant dengue NS1 antigen and tested. The absorbance was read in the ELISA reader at 450 nm. *NC* Negative control, *PC* Positive control.

Based on our previous experiment in unfed *Ae. aegypti* mosquitoes, we were further interested in checking whether blood constituents may influence the sensitivity of the kit for detection of rDENV-2 NS1 spiked with blood-fed *Ae. aegypti* mosquito pools. It was observed that blood-feeding does not necessarily interfere with the assay sensitivity as the kit displayed similar absorbance values in unfed and blood-fed mosquito pools. This evinced that the kit is equally sensitive for detection of rDENV-2 NS1 antigen in the blood-fed mosquito (Fig. 6).

### Detection of NS1 antigen of DENV infected human serum in *Ae. aegypti* mosquito pools

**Figure 7:**
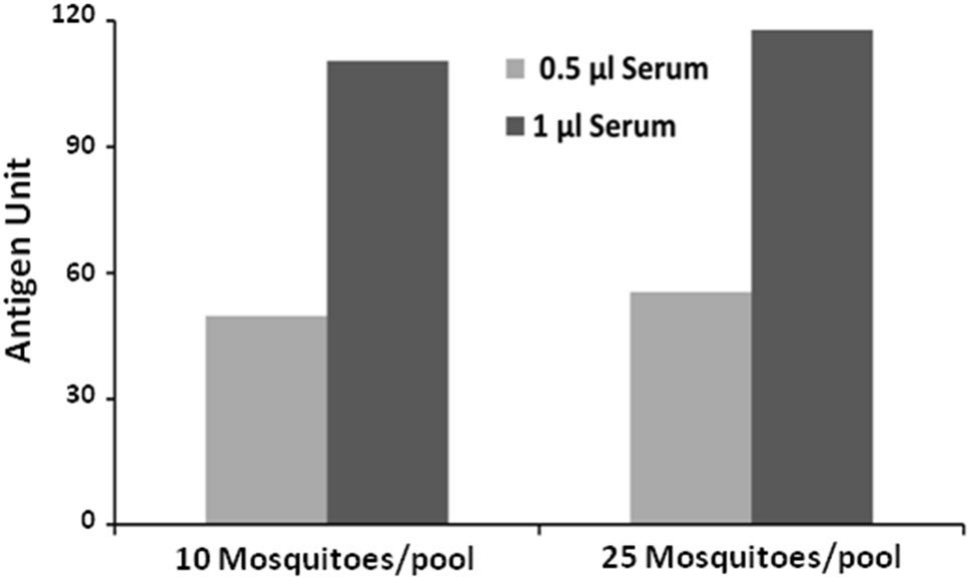
Detection of NS1 antigen in *Aedes aegypti* mosquito homogenate spiked with dengue patient serum. The dengue confirmed patient serum was spiked with mosquito homogenate and ELISA was performed. OD measured at 450 nm was interpreted in terms of antigen unit as per manufacturer’s instructions.

The Microlisa kit in DENV infection confirmed patient was tested before spiking the serum with the mosquito pools. The serum sample tested at both the dilutions (1:50 and 1:100) displayed OD > 6, which is beyond the limit of detection of the ELISA reader (Fig S3). The same serum sample was used to spike in the *Aedes aegypti* mosquito pools to simulate NS1 protein secretion in the mosquitoes. It was observed that the kit is highly sensitive to detect the NS1 antigen of the serum sample spiked in the *Ae. aegypti* mosquito pools (Fig. 7).

## Discussion

Current methods of DENV detection in the field-caught mosquitoes suffer from one or the other limitations. To overcome such limitations, commercially available NS1 antigen-based ELISA and Rapid diagnostic tests have been tested. The possible use of Dengue NS1 Ag Strip (BioRad Laboratories) for field surveillance of infected dengue vectors was attempted in Singapore^15^. PanBio dengue early ELISA kit was tested for surveillance of DENV infected mosquitoes in Australia and India^14,20^. In another study, a commercially available Platelia Dengue NS1 Ag kit was evaluated to detect DENV in infected *Ae. aegypti* and found to be more convenient to test mosquitoes with DENV infection^21^. SD Bioline NS1 Antigen kit was used to test dengue antigen in adult Aedes mosquitoes in Selangor, Malaysia^18^. Our group has recently evaluated the DENV Detect™ NS1 ELISA kit (InBios International, Inc.) to detect rDENV-2 NS1 protein in *Ae. aegypti* mosquitoes (manuscript communicated to J Vector Borne Dis). As an extension to this investigation, we tested the utility of Dengue NS1 Ag Microlisa (J. Mitra & Co. Pvt. Ltd) to detect rDENV-2 NS1 spiked with *Ae. aegypti.* The Microlisa kit is highly sensitive and specific (99.5% and 100% respectively as mentioned in the kit insert) for diagnosis of dengue. Therefore, the kit was explored for detection of rDENV-2 NS1 antigen as well as dengue NS1 antigen positive sera in pools of *Ae. aegypti* mosquito samples.

Since dengue is caused by four serotypes of dengue virus (DENV1 to DENV4), it is necessary that the kit should be sensitive for detection of all forms of dengue NS1 antigen. Therefore, we tested NS1 antigen of all serotypes of DENV. The kit detected all the DENV serotypes indicating that it is highly sensitive for detecting any serotype of DENV. As NS1 antigen is common to all flaviviruses, the kit may show the cross-reactivity of DENV NS1 antigen with other flaviviruses, leading to false-positive results. Therefore, the Microlisa kit was tested with recombinant NS1 antigens of four flaviviruses such as JEV, TBEV, WNV, and YFV. The major purpose of testing NS1 antigens of flaviviruses other than dengue virus was to check the specificity of the test (100% as mentioned by the manufacturer). We observed that the kit could not detect NS1 antigen of JEV, TBEV and WNV suggesting that the kit is highly specific for detection of dengue. Although, NS1 antigen of YFV cross-reacted at 750 pg and 250 pg antigen concentrations, it is noteworthy that India is free from yellow fever virus^22^.

Based on these observations, we tested rDENV-2 NS1 antigen detection limit of dengue NS1 Ag Microlisa Kit and found that the kit is highly sensitive as it can detect the NS1 antigen in femto gram level. A spiking experiment was designed to check the sensitivity of the kit to detect rDENV-NS1 in *Ae. aegypti* mosquito pools (25/pool) employing two procedures. It was observed that the kit was sensitive to detect the rDENV in these mosquito pools having minimum 2.5 pg rDENV-2 NS1 antigen. This suggests that the kit could be used for the detection of NS1 antigen even up to pico gram levels. Though the antigen unit at 25 pg and 7.5 pg was higher for the 1st procedure, the antigen units remained almost the same at low antigen concentration (2.5 pg). This indicated that NS1 antigen could be detected in field-caught mosquitoes. It was also observed that the kit was equally sensitive to detect rDENV-2 NS1 antigen in both the mosquito pools.

Further, to simulate the condition of detection of NS1 antigen in the dengue infected human blood-fed mosquitoes, the serum sample of a dengue infected patient was spiked with the *Ae. aegypti* mosquito pools (10 and 25/pool). It was observed that the kit was highly sensitive to detect the NS1 antigen in both the pool sizes. This observation indicates that the kit can be used for the detection of DENV infection in the field-caught *Ae. aegypti* mosquitoes also.

A recent study on the comparative assessment of commercial ELISA tests used for DENV diagnosis in India showed that J. Mitra Dengue NS1 Ag Microlisa was more efficient than WHO-approved Panbio Dengue Early ELISA for the detection of all the four DENV serotypes in India^23^. Although like other commercial dengue NS1 antigen diagnostic kits, the Microlisa kit is also intended for dengue diagnosis in humans, this kit has not been explored for detection of NS1 protein antigen in DENV infected *Ae. aegypti* mosquitoes. In the current study, we attempted for the first time to test this kit for detection of rDENV-2 NS1 as well as NS1 antigen from the serum of DENV infected patients in pools of *Ae. aegypti* mosquitoes. In both cases, the kit was found to be highly sensitive to detect dengue virus NS1 antigen. This investigation warrants further studies on DENV infected *Ae. aegypti* mosquitoes and field-collected mosquitoes towards exploring the utility of this kit as a tool for the dengue vector surveillance.

## Supplementary Information

Supplementary Information.
