# Supplementary material for: Dengue NS1 antigen kit shows high sensitivity for detection of recombinant dengue virus-2 NS1 antigen spiked with Aedes aegypti mosquitoes
Source: Sci Rep. 2021 Dec 8;11:23699. doi: 10.1038/s41598-021-02965-x (PMC8655051; doi:10.1038/s41598-021-02965-x)
Supplement: Supplementary file 1 — Supplementary Information. [file 41598_2021_2965_MOESM1_ESM.pdf]

Dengue NS1 antigen kit shows high sensitivity for detection of recombinant dengue virus-2 NS1 antigen spiked with *Aedes aegypti* mosquitoes

Philip Raj Abraham[1]* Bharathy R[1], Pradeep Kumar N[2] & Ashwani Kumar[3]

[1]Unit of Omics, ICMR-Vector Control Research Centre, Puducherry 605006, India

[2]ICMR-Vector Control Research Centre Field Station, Kottayam – 686002, Kerala, India

[3]ICMR-Vector Control Research Centre, Indira Nagar Puducherry 605006, India

***Corresponding author**

Dr. Philip Raj Abraham, Ph.D

Scientist-B and Head

Unit of Molecular Epidemiology

ICMR-Vector Control Research Centre

Puducherry 605006, India

Email: microphilip_14@rediffmail.com

Telephone: +91 0413 227 2396

Cell: +91-7569765907

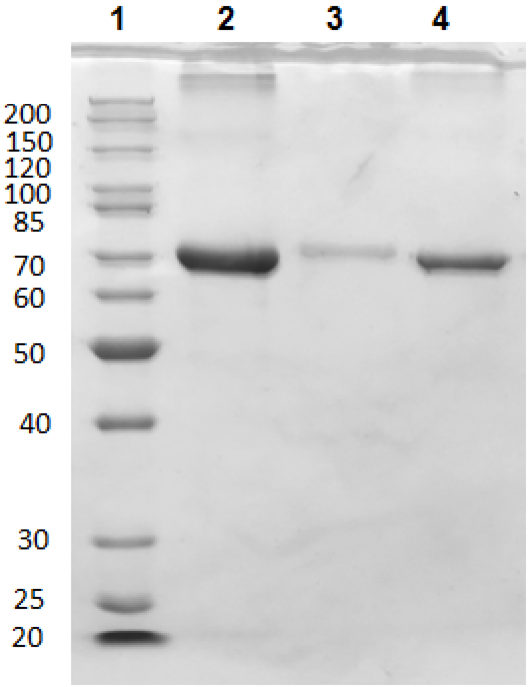

Supplementary Figure 1: SDS-PAGE of Dengue NS1 Ag Microlisa kit controls. The control samples were run along with the unstained protein marker on 12% SDS-PAGE and stained with Coomassie Brilliant Blue. 1. Protein marker 2. Positive control 3. Enzyme conjugate 4. Calibrator control.

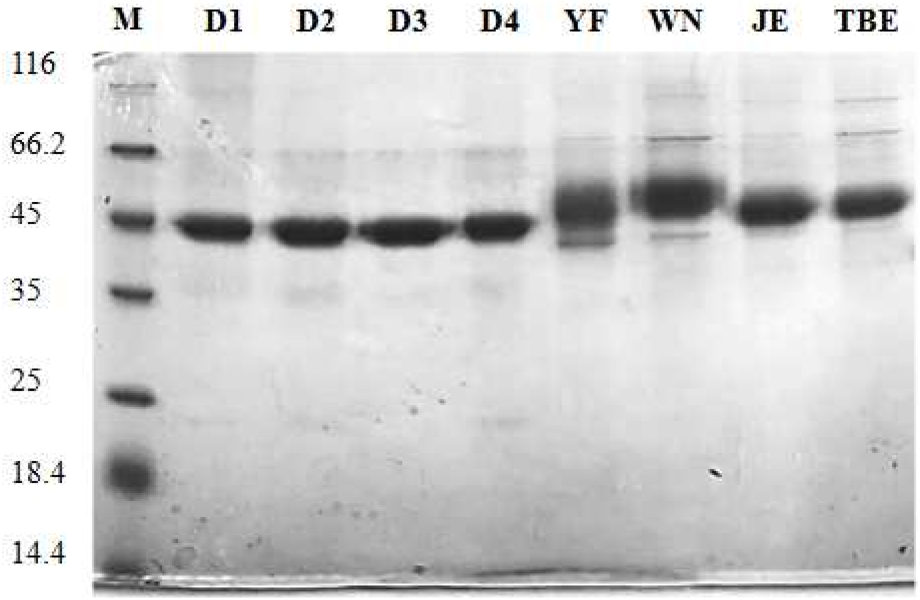

Supplementary Figure 2: SDS PAGE of NS1 antigen of flaviviruses. NS1 proteins of flaviviruses were run along with the unstained protein marker on SDS-PAGE (12%) and stained with Coomassie Brilliant Blue. M – Protein marker, D1 – Dengue serotype 1, D2 - Dengue serotype 2, D3 - Dengue serotype 3 , D4 - Dengue serotype 4, YF – Yellow Fever virus, WNV - West Nile virus, JE – Japanese encephalitis virus, TBE – Tick-borne encephalitis virus.

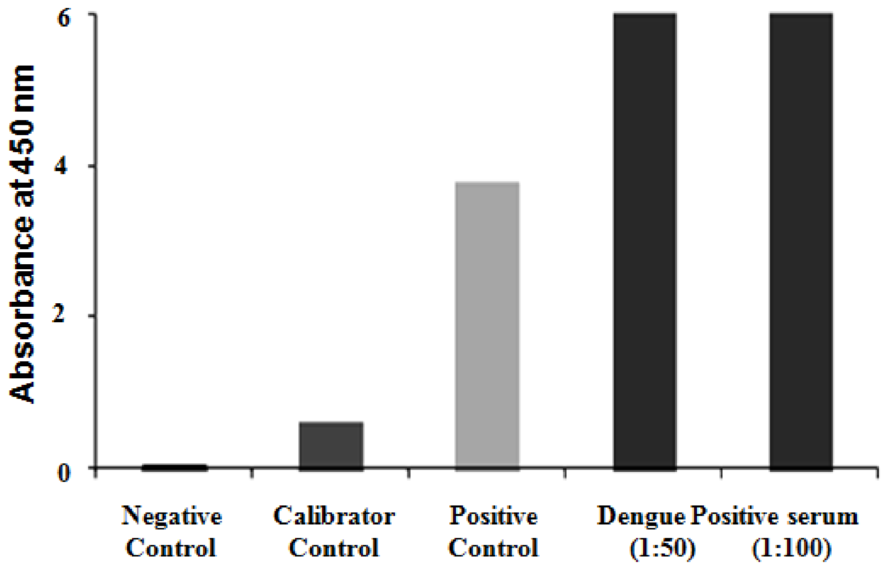

Supplementary Figure 3: Detection of NS1 antigen in dengue patient's serum. Dengue infected patient serum was added to the Microlisa plate followed by enzyme conjugate and incubated. Absorbance at 450 nm was read after addition of stop solution.
